# 

**Published:** 1876-12

**Authors:** 


### INDEX.

### ORIGINAL COMMUNICATIONS.

Acid, Salicylic. *D. T. Nelson* ........ 876
Adenia, with Cases. *N. A. Drake* ........ 499
Amnion, Dropsy of the; and Emphysematous Fœtus. *J. Schneck* ....1103
Amnion, Dropsy of the. *W. H. Byford* ........ 1
Appendix Vermiformis, Perforation of. *C. W. Earle* ........ 121
Blood Pressure in the Left Ventricle and Aorta. *H. Gradle* ........ 673
Bowel Affections of Infants. *E. W. Sawyer* ........ 13
Brain, Concussion of the. *F. W. Mercer* ........ 609
Cataract, Jaeger's Operation for. *L. Ware* ........ 496
Chloroform, Ether and Morphia in Obstetrics. *Ph. Adolphus* ........ 306
Chloral Hydrate Poisoning. *H. W. Meyer* ........ 972
Cholera, Report on. *Drs. Danforth and Hyde.* ........ 418
Cholera viewed from a new standpoint. *H. R. Rogers* ........ 289
Cinchonidia Sulphate. *E. H. Purdum* ........ 504
Convulsions, a Case of Clonic. *C. A. Rood* ........1085
Croton Chloral Hydrate in Photophobia. *P. Owen-Jones* ........ 776
Cystocele, Case of. *R. M. Lackey* ........ 594
Diagnostic Syllabus of Inflammation in Uterus and Vagina. *J. H. Etheridge* ........ 812
Diagnostic Syllabus of Roetheln, Scarlet Fever and Measels. *J. H. Etheridge* ........ 312
Disinfecting Oven in Mercy Hospital. *E. Andrews* ........ 325
Early Medical Chicago. *J. N. Hyde* ........ 193
Empyema, Treatment of. *W. H. Vittum* ........ 869
Epilepsy, Cases of Excentric. *W. R. McMahan* ........ 397
Epilepsy, Therapeutics of. *A. McLane Hamilton* ........1057
Ergot as a Galactifuge. *C. H. Leonard* ........ 322
Erysipelas, Gangrenous. *M. A. McClelland* ........ 404
Ether, Death from. *E. L. Holmes* ........ 409
Etiology, the Study of. *N. S. Davis* ........ 679
Eyeball, Rupture of. *F. C. Hotz* ........ 97
Fistula, Case of Vesico-Vaginal. *A. B. Newkirk* ........ 508

Forceps, A new Obstetric. *E. W. Sawyer* ... 46
Fracture, Intracapsular of Neck of Femur. *T. J. Maxwell* ... 401
Fracture of the Skull, Comminuted. *J. W. Burns* ... 865
Fragilitas Ossium. *W. Blanchard* ... 7
Hemoptysis Vicarious. *J. S. McCord* ... 503
Hohmann, Kath. *E. W. Sawyer* ... 392
Melancholia, Suicidal, a Case of. *D. R. Brower* ... 690
Microscope, In daily Practice. *I. N. Danforth* ... 116
Monstrosity, Case of. *C. P. Simon* ... 719
Mortality of Surgical Operations in the Upper Lake States. *E. Andrews* ... 577, 700, 891, 974, 1087
Os Uteri, Rigidity of ... 717
Ovarian Dermoid Tumors. *W. H. Byford* ... 299
Ovarian Tumors, Tapping of. *M. B. Wright* ... 414
Ovarian Tumor, Spontaneous Cure of. *O Wright* ... 607
Ovariotomy in a Patient Sixty-Five Years of Age. *C. Richards* ... 769
Ovariotomy, Two Cases of. *A. R. Jackson* ... 779
Pericarditis, Acute. *D. A. K. Steele* ... 599
Phlegmasia Dolens, as a Symptom of Intra-Abdominal Cancer. *H. M. Lyman* ... 318
Pile Surgeons, Itinerant. *E. Andrews* ... 887
Pigmentary Deposits in the Cortical Substance of the Encephalon. *A. W. Hagenbuch* ... 774
Placenta Prævia, True Site and Cause of. *J. Bartlett* ... 967
Polypi, Intra-Uterine. *A. R. Jackson* ... 104
Repair, Experimental Study of the Process of. *I. N. Danforth* ... 1071
Report on Progress of Chemical Investigation. *H. M. Lyman* ... 488
Spine, Case of Injury of. *E. W. Lee* ... 889
Superior Strait, Abnormally Large. *J. S. Knox* ... 385
Syphilis, Etiology of Hereditary. *F. R. Sturgis* ... 514
Transfusion of Defibrinated Human Blood. *M. W. Wood* ... 481
Tumor of the Sterno-Mastoid Muscle. *A. R. Jackson* ... 961
Uterus, Fibroid Tumors of. *J. B. Crandall* ... 686
Uterine Fibroid Tumors Treated by Ergotine. *D. A. K. Steele* ... 610
Uterus, Subperitoneal Fibroid Tumor of the. *R. S. Sutton* ... 1079
Uterine Cancer, Supposed Case of. *E. W. Jenks* ... 772
Uterine Displacements, Mechanics of. *A. H. Scott* ... 788

### TRANSLATIONS.

Fiori, G. M. A Case of Cerebral Syphilis ... 1018
Hubert, E. The Action of the Forceps ... 649, 845
Mooren, A. Acute Cyclitis ... 88
Rohlf. Medicine and Fine Arts ... 569
Rohlf. Medicine and Poetry ... 571

### SUMMARY.

### I. Anatomy and Physiology.

A Method of Observing the Circulation in the Frog's Lungs. *Holmgren* 70
Congenital Myosarcoma of the Kidneys. *Cohnheim* 141
Force of Ciliary Motion. *Bowditch* 953
Lymphatic Vessels of the Joints. *Tillmans* 141
New Sphygmograph. *Keyt* 142
Physiological Action of Lobelina. *Ott* 143
Theory of the Dicrotic Pulse. *Henry* 163
Urine of New-born Infants. *Parrot and Robin* 331
Vaso-motor Nerves and their Action. *Masius and Vanlair* 71

### II. Practical Medicine.

Aneurism, Intra-cranial, recovery under large doses of Iodide of Potassium. *W. E. Humble* 459
Atrophy, Progressive, Muscular. *W. Alexander* 458
Alcohol and Acids, Influence of upon Coagulation of the Blood. *Oré.* 253
Anus, Fissure of in Infants, Diagnosis of. *Marbaux* 767
Aspirator, Simplified. *Otis* 345
Aorta, Obliteration of. *Lüttich* 252
Anæmia, or Anæmatosis, Progressive, Pernicious. *Pepper* 65
Bronchitis, Acute, Fibrinous, with Tube Casts. *E. D. Worthington* 954
Burns, Disorders of Vascular System in. *Coustou* 954
Chorea, The Cause of. *Baker* 1135
Paraplegia, Pseudo, a New Form of. *T. G. Stewart* 344
Catarrh, Post-nasal. *B. Robinson* 150
Chorea, Cardiac. *Boyer* 341
Croup, Hydrotherapeusis of. *Winternitz* 659
Cirrhosis of Liver in Child. *T. D. Griffiths* 253
Diarrhœa, Intermittent. *Rothe* 1050
Diagnosis, Differential, of Hysteria and Epilepsy. *Delasiaune* 342
Diphtheria, Affecting the Nipples. *T. L. Wright* 662
Dislocation of Shoulder, Anæsthesia of Deltoid Region in. *Th. Anger.* 348
Dysentery, Chronic — Local Treatment. *T. G. Thomas* 149
Epileptic Phenomena from Cerebral Tumor. *De Bonis* 1137
Etherization of Young Children. *Tripier* 1051
Epilepsy, Chronic, Observations on. *A. McL. Hamilton* 1047
Fibrinous Bronchitis, with Tube Casts 954
Foreign Bodies in Brain, with Suicidal Intent. *W. B. Carpenter* 856
Fatty Degeneration of Liver and Kidneys from Burn. *Canston* 456
Fever, Typhoid, from Drinking Water. *Brown* 66
Gangrene of Limbs in Typhoid Fever. *Valette* 857
Glossitis, Idiopathic. *Wingate* 1135
Gall-Stones, Rare Case of, Discharged through the Side. *D. Perley* 955

Hair, Hygiene of. *Bazin* ........ 1052
Heart Disease, Antagonism between and Tuberculosis. *Peter* ........ 457
Hyperæsthesia, Thoracic, Relation of to Acute Tuberculosis. *Bouchut.* 458
Hæmorrhage, Internal, in Typhoid Fever. *Th. Plagge* ........ 258
Hysteria, Simulating Progressive Locomotor Ataxia. *W. H. Webb* ........ 257
Heart, Rupture of. *R. E. Van Giesen* ........ 255
Headache, Frontal. *Brunton* ........ 65
Intermittent Broncho-Pneumonia. *Bourgade* ........ 341
Intestine, Obstruction of, due to Biliary Calculus. *Martin and Bronardel* ........ 341
Intussusception in an Infant. *F. L. Haynes* ........ 459
Lightning, Death by. *B. Pietro* ........ 345
Lymphatic Radicles, Dilatation of. *C. Hanfield Jones* ........ 67
Liver, Capillary Puncture of, followed by Urticaria. *Laveran* ........ 768
Lung, Encephaloid Disease of. *G. G. Tyrrell* ........ 152
Leucocytes, Diapedesis of. *N. B. Siger* ........ 858
Maternal Impressions. *Waddel* ........ 1136
Mortality of Mariners, etc. *E. Holden* ........ 346
Mumps, Relation to Eruptive Fevers. *Calin* ........ 457
Milk Diet in Albuminuria of Pregnancy. *Tarnier* ........ 661
Microscope, Ready Method of preparing Sections for. *J. Stevenson* ........ 347
Nervous System, Relation of Teeth to Disturbances of ........ 460
Nursing and Weaning of Infants. *Magne* ........ 1139
Otis' Aspirator Simplified ........ 345
Peritonitis, Remarkable Case of. *E. J. Abbott* ........ 661
Pneumonia, Infectious. *A. W. Blyth* ........ 344
Paralysis Agitans, Lesions in. *R. F. Lautenbach* ........ 1047
Phthisis, Menses of Patients in. *Ladmiral* ........ 955
Phymosis, Diabetic. *Niepee* ........ 1048
Paralysis, Facial, Diagnosis of Seat of Lesion of. *S. G. Webber* ........ 1046
Pica, Case of. *A. N. Gould* ........ 662
Purpura, Neurotic. *Tyrrell* ........ 1049
Pneumatometry. *Elsberg* ........ 64
Poisoning by Coal Gas. *R. Dexter* ........ 1047
Pertussis, Abortive Treatment by Quinine. *R. K. Hinton* ........ 955
Pericardium, Paracentesis of. *Burder* ........ 460
Paralysis, General, Unique Anatomical Appearance in. *W. H. DeWitt.* 459
Pulmonary Disease, Pneumatic Treatment of. *Schnitzler* ........ 660
Pertussis, Sedative Solution in. *Massy* ........ 660
Pneumonia, Endemic of Miasmatic Infections. *W. B. Rodman* ........ 256
Pulmonary Collapse. *H. A. Dubois* ........ 151
Pigment, Formation of in Melanotic Sarcoma. *Gussenbauer* ........ 252
Pulse, Functional Infrequency of. *Austin Flint* ........ 254
Pneumonia, Pathology of. *J. Dreschfeld* ........ 254
Rheumatism and Traumatism. *Verneuil* ........ 343
Rheumatism treated with Salicylate of Soda. *Clarke* ........ 1134
Rheumatism, Acute, Cure of by Salicylic Acid ........ 253

Re-vaccination in Cincinnati. *W. B. Davis* 258
Râle, Crepitant, Nature and Production. *W. H. Workman* 1045
Râle of the Buccal Cavity. *Galvagni* 765
Rectum, Mass of Cherry Stones in. *W. H. Wescott* 347
Salicylic Acid in Microscopy. *Edwards* 1049
Strangulation of Ileum by Mesentery. *J. Bradley* 148
Stomach, Fork in, Gastrotomy. *Labbe* 764
Sleep of the Aged. *Popelauer* 660
Tænia, Slate-colored. *Laboulbène* 342
Tongue, Congenital Hypertrophy of, Relieved by Galvano-Cauterization. *Valerani* 1048
Teeth, Relation of to Disturbances of Nervous System 460
Traumatism and Rheumatism. *Verneuil* 343
Typhoid Fever, Discordance of Pulse and Temperature in. *Desnos* 1048
Throat and Air Passages, Follicular Disease of and Pathology of. *B. Robinson* 150
Tongue, Swelling of with Profuse Salivary Flow. *W. Carson* 858
Tetanus, Rheumatic, Cured by Salicylic Acid. *Wunderlich* 1044
Tetanus, Cure of by Mechanical Measures. *Calastrie* 856
Variola, Treatment of by Exclusion of Solar Light. *Waters and Gaddesden* 766
Vertigo, Auditory. *Gowers* 1138
Vomiting of Curious Worm. *Sager* 346

### III. Surgery.

Ainhum. *Camuset* 1053
Amputation in a Diabetic Patient. *Verneuil* 659
Anæsthesia, Bonwill's Method of. *A. Hewson* 658
Anæsthetization during Sleep. *Cluness* 335
Aneurism, Intra-cranial. *W. E. Humble* 260
Aneurism of the Aorta successfully Treated. *Thos. Annandale* 260
Aneurisma Arterioso-venosum in the Orbit. *Rivington* 53
Antiseptic Treatment, The Operation for Hydrocele under. *Volkmann* 332
Antiseptic Treatment, Results of. *A. Bardeleben* 58
Calculus, New Sound and Extractor for. *Waitz* 333
Calculi, Salivary. *Pirondi* 336
Caoutchouc in Surgery. *Tarogenne* 859
Carbolic Acid for Poisonous Wounds. *Klamann* 145
Carbuncular Disease treated by Subcutaneous Injections. *Raimbert* 656
Catheter, Hydrostatics of the. *Sommerville* 262
Chloroform Asphyxia, How to Prevent. *J. Fleiberg* 55
Diphtheria, Treatment of. *Theo. Schueler* 55
Encephaloid Cancer. *C. V. Moltram* 58
Epithelioma of the Tongue. *Trélat* 657
Female Bladder, Hair-pin Extracted from the. *Panas* 337
Fistula, Recto-urethral. *Benningham* 263

Fracture of Neck of Femur. *Welmart* .......... 336
Fracture of the Base of the Cranium. *Voltolini* .......... 956
Fractures, Treatment of. *Schwab* .......... 261
Hernia, Radical Cure of. *Nussbaum* .......... 455
Hospital Gangrene .......... 861
Hydrocele, Operated under Antiseptic Treatment. *Volkmann* .......... 332
Hydrocele Cured by Carbolized Injections. *Rahn* .......... 262
Ileo-Femoral Triangle, Diagnostic Value of the. *Thos. Bryant* .......... 859
Injections, Parenchymatous. *Wilde* .......... 259
Intussusception, Abdominal Section for. *H. Marsh* .......... 334
Larynx, Extirpation of the .......... 55
Luxation of the Thumb backward. *Farabeuf* .......... 453
Nerve-stretching for Tetanus. *Vogt* .......... 1140
Nerves, Excision and Reunion of. *Braun* .......... 1053
Nasal Diseases treated by Bougies. *Catti* .......... 1143
Osteitis of Pearl Turners. *Gussenbauer* .......... 57
Phymosis, Operation for. *Anger* .......... 336
Resection of Superior Maxilla. *Letiévant* .......... 1141
Rhinoscopy. *Ph. S. Wales* .......... 56
Rhinoplastic Operation, New. *Hardie* .......... 658
Salicylate of Soda for Rheumatism. *Clarke* .......... 1134
Salicylic Acid as a Dressing for Wounds. *Callender* .......... 145
Skin-grafting, A Remarkable Case of. *Reverdin* .......... 455
Staphylorraphy, New Method of. *Schoenborn* .......... 957
Subcutaneous Injections in Carbuncular Disease. *Raimbert* .......... 656
Sudden Death, Fatty Embolism as a cause of. *Czerni* .......... 452
Tarsal Bones, Removal of the. *Dawson* .......... 146
Tendons, Sutures of. *Tillaux* .......... 146
Tendons United by Sutures. *Fillauz* .......... 656
Tetanus Cured by Nerve-stretching. *Vogt* .......... 1140
Tracheal and Laryngeal Stenosis. *Thaou* .......... 1142
Toe Nail, Treatment of In-growing. *Bertheraud* .......... 957
Varicocele, Treatment of. *Ravoth* .......... 147

### IV. Obstetrics and Gynæcology.

Abortion and Hæmorrhage. *Pallin* .......... 463
Asphyxia Neanatorum, Treatment of. *Budin* .......... 354
Asthma, Uterine. *Warring* .......... 270
Abortion, Management of. *Hazzard* .......... 157
Abortion, Legal Aspect of. *Leblorde* .......... 50
Bladder, Irritable, Treated by Dilatation. *Hewetson* .......... 273
Conception, Forty Hours after Abortion. *Sparkman* .......... 666
Cystitis, Subacute, after Parturition. *Richardson* .......... 542
Cæsarian Section, Cases of. *Belluzzi and Testa* .......... 353
Cord, Best Time for Ligature of. *Budin* .......... 355
Cervix Uteri, Use of Caustics in Diseases of. *Gilt* .......... 271
Confinements, Management of. *Hime* .......... 49

Carcinoma Uteri, Chloral in. *Fleischer* ........ 52
Endometritis, Chronic Corporeal, Treated by Pressure. *Reamy* ........ 355
Fœtus, Death of from Knot in Cord before Confinement. *Canivet* ........ 461
Fibroids of Uterus, Unusual Terminations of. *Parvin* ........ 268
Fœtal Heart heard early in Pregnancy. *Underhill* ........ 156
Gestation Continued three months after Rupture of Membranes. *Rains* 156
Gestation, Case of Prolonged. *Wells* ........ 158
Hydatids of Womb. *McClelland* ........ 462
Injections, Evil Effects of. *Padlock* ........ 272
Lactation, Induced. *Gilbert* ........ 463
Mumps, Metastasis in Women. *Damorest* ........ 947
Mastitis, Eclampsia from. *Panthel* ........ 666
Menstruation, Early Case of. *Anon.* ........ 166
Menstruation and Law of Monthly Periodicity. *Goodman* ........ 166
Ovulation without Menstruation. *St. Germain* ........ 264
Ovulation and Menstruation. *Williams* ........ 161
Pregnancy, Triple. *Leriche* ........ 947
Pregnancy, Harmlessness of Grave Operations in. *Nicaise* ........ 750
Puerperal Fever Prophylaxis. *Bischoff* ........ 541
Pregnancy, Tubal. *Albu* ........ 461
Placenta and Hæmorrhage from Abortion ........ 463
Pregnancy extending 306 days. *Graves* ........ 464
Perineum, Fibroid Tumor of. *McLellan* ........ 356
Pessary, Cotton. *Page* ........ 274
Pessary, Productive of Paralysis. *Gervis* ........ 159
Singultus, Uterine. *Fritsch* ........ 356
Twins with Different Birthdays. *Kollock* ........ 667
Uterine Affections treated by Hydrotherapy. *Derivaux* ........ 948
Uterus, Gravid, Laceration of. *Thompson* ........ 663
Ulcerations, So-called, of Uterus. *Wing* ........ 543
Uterus, Rupture. *Fuller* ........ 463
Uterus, Gravid, Physiological Cause of Muscular Contraction of. *Mays* 464
Uterine Disease, Malignant, Tolerance of Sedatives in. *Day* ........ 267
Vagina Formed without Cutting Instruments. *Le Fort* ........ 947
Vagina, Cysts of. *Dupuy* ........ 750
Vaginismus, Iodoform in. *Tarnier* ........ 51

### V. Therapeutics.

Alimentation, Hypodermically. *Krueg* ........ 275
Abscess, Opened with Carbolic Acid. *Bergonzini* ........ 276
Amyl Nitrite, Uses of. *Pick* ........ 756
Alcohol as a Dressing. *Boslee* ........ 1043
Air as an Anæsthetic. *Keyser* ........ 1044
Atropia *versus* Hydrocyanic Acid. *Jackson* ........ 1134
Bromohydric Acid. *Fothergill* ........ 1133
Bisulphide of Carbon for Cancer of Stomach. *Whitaker* ........ 1130
Blood, Lambs, Subcutaneously, in Melancholia. *Voisin* ........ 350

Bones, Dressing for. *Smith* 352
Bromide of Potassium, Local Use of. *Coomes* 949
Cinchonidia, Sulphate of. *Batley* 1133
Creasote for Tapeworm. *Brickwell* 152
Camphor in Erysipelas. *Revillout* 155
Chloral Anæsthesia of Infants. *Bouchut* 155
Carbolic Acid for Opening Abscesses. *Bergonzini* 276
Coffee *versus* Malaria. *Holland* 276
Chloroform Narcosis and Nelaton's Restoration. *Smith* 277
Choleate of Soda in Gall Stones. *Dabney* 669
Croton Chloral. *Emmert* 757
Cold *versus* Tartar Emetic. *Washington* 758
Carbolic Acid in Diabetes 760
Cold in the Head, Treament of. *Ferrier* 761
Chloroform in Anal Fissure. *Nicaise* 762
Convulsions Treated by Posture. *Brown* 763
Cold in Abdominal Inflammations. *Eads* 1041
Chloral in Carcinoma Uteri. *Fleischer* 52
Chloral Plaster for Neuralgia 1133
Diarrhœa, Salicine in. *Vail* 68
Diabetes Treated with Glycerine. *Jacobs* 348
Diphtheria, Treatment of 753, 760
Dysentery, Chronic, Treatment of. *Dills* 759
Diabetes Treated with Carbolic Acid 760
Diphtheria Treated with Sodic Hyposulphite. *Tamborlini* 763
Diphtheria, Turpentine in. *Peabody* 1041
Erysipelas Treated with Camphor. *Revillout* 155
Erysipelas, Treatment of. *Yandell* 349
Ergot as an Antipyretic. *Hayem* 352
Epistaxis, Ergot in. *St. George* 549
Electricity, Uses of. *Anon* 550
Electrolysis in Tumors. *Althaus* 752
Enteric Fever, Sulphurous Acid in. *Botkin* 759
Goats' Milk, Poisoning by. *Anon* 277
Glycerine in Diabetes. *Jacobs* 348
Grindelia Robusta, Action of. *Rademaker* 466
Gall Stones, Sodic Choleate in. *Dabney* 669
Gelseminun, Action of. *Hertzka* 762
Gonorrhœa, Quinine Injections in. *Nauth* 1041
Glycerine, Toxic Properties of. *Beaumetz* 1043
Hæmoptysis, Poultices in. *Mussy* 756
Hydrophobia, Xanthium Spinosum in. *Grzyvala* 757
Hyposulphite of Soda in Diphtheria. *Tamborlini* 763
Hyoscyamia for Vomiting in Pregnancy. *Pitois* 1130
Infants, Medicine to, through Mother's Milk. *Lewald* 69
Intermittents, Chronic, Spider Web in. *Jones* 667
Iodoform, Odorless 761

Insolation Treated with Quinine Hypodermically. *Hall* 762
Inflammation, Abdominal, Cold in. *Eads* 1041
Iodoform in Vaginismus. *Tarnier* 51
Jaborandi, Action. *Dumas* 549
Mint in Neuralgia. *Delioux* 69
Malaria *versus* Coffee. *Holland* 276
Melancholia Treated with Lamb's Blood Subcutaneously. *Voisin* 350
Neuralgia, Mint in. *Delioux* 69
Nitro-Glycerine, Action of. *Minor* 761
Opium, Antidotes to. *Holland* 274, 465
Odontalgia, Treatment of. *Guild* 465, 756
Oxygen Gas. *Heard* 1134
Prussic Acid, Action of. *Boehm and Knie* 350
Poison, General Antidote to. *Jeanneal* 351
Pertussis and Etherization. *Powell* 465
Picrotoxine, Action of. *Gubler* 550
Pomegranate Bark in Tape Worm. *Freeman* 668
Poultices in Hæmoptysis. *Mussy* 756
Quinine in Surgery. *Verneuil* 549
Qunine in Insolation. *Hall* 762
Quinine in Gonorrhœa. *Nauth* 1041
Quinine Bromohydrate. *Gubler* 1132
Quinine, Elimination of. *Albertoni and Ciotto* 1129
Quinine, New Use of 1131
Remittent Fever, Senna in 277
Salciylic Acid for Perspiration of Feet. *Kuester* 1133
Salicine and Salicylic Acid in Rheumatism. *Madagan* 1132
Salicine in Diarrhœa. *Vail* 68
Spleen, Tumefaction, Treatment of. *Mosler* 70
Stomach Pump, Treatment with. *Schleip* 153
Senna in Remittent Fever. *Anon* 277
Salicylic Acid in Rheumatism 278, 349, 465, 547, 756
Scilla, Action of. *Husemann* 278
Salicylic Acid in Foul Breath. *DaCosta* 350
Scrofulous Sores, Treatment of. *McLennan* 351
Spider Web in Chronic Intermittents. *Jones* 667
Salicylic Acid as an Antipyretic. *Ewald* 754
Sulphurous Acid in Enteric Fever. *Botkin* 759
Salicylic Acid, Action of 949, 1042
Tape Worm Treated with Creosote. *Brickwell* 152
Tape Worm Treated with Pomegranate. *Freeman* 668
Tumors, Electrolytic Treatment of. *Althaus* 752
Tobacco, Anaphrodisiac Properties of. *Martin* 760
Turpentine in Tonsillitis and Diphtheria. *Peabody* 1041
Tonsillitis, Turpentine in. *Peabody* 1041
Water, Subcutaneous, in Pain. *Dessan* 755

### VI. Ophthalmology and Otology.

Atrophy of the Eyeball, Artificial. *Vidor* ........ 959
Blindness, Daily Recurring. *Koenigstein* ........ 339
Castor Oil for Dissolving Atropia. *Green* ........ 340
Cornea, Tattooing of. *Howe* ........ 338
Diabetes Mellitus, Affections of the Eye due to. *Leber* ........ 862
Eyeball, Artificial Atrophy of. *Vidor* ........ 959
Middle Ear, Inflation of the. *Gruber* ........ 143
Nasal Douche, Injurious Effects of. *Shaw* ........ 958
Neuro-retinitis by Lightning. *Brière* ........ 862
Nystagmus, Miner's. *Taylor* ........ 54
Ophthalmoscopy in Cerebral Lesions. *Bouchut* ........ 861
Ophthalmia Neonatorum. *Ayres* ........ 340
Otorrhœa, Treatment of. *Politzer* ........ 1054
Otorrhœa, Operative Treatment of. *Wolf* ........ 1055
Otorrhœa, Styptic Cotton for. *Morton* ........ 144
Otorrhœa, Salicylic Acid for. *Chisolm* ........ 144
Orbit, Anenrism in the. *Rivington* ........ 53
Tattooing of the Cornea. *Howe* ........ 338

### VII. Dermatology.

Alterations of the Color of the Hair and Skin after Scarlatina. *Wallenberg* ........ 670
Anatomy of Lupus Erythematodes. *Geber* ........ 671
Biniodide of Mercury in Superficial Lupus. *Guibout* ........ 952
Case of Argyria. *Riemer* ........ 170
Collodion for Freckles ........ 173
External Application of Carbolic Acid in Cutaneous Diseases. *Berndgren* ........ 173
Feigned Erythema Gangraenosum. *Fox* ........ 171
Generalized Melanosis. *Bergeron* ........ 170
Herpes Zoster Gangraenosus Recidivus. *Kaposi* ........ 669
Pellagra. *Béhier* ........ 169
Proofs of Dartrous Diathesis Furnished by Comparative Pathology. *Mégnin* ........ 167
Psoriasis of the Tongue. *Trélat* ........ 670
Rheumatismal Bullous Erythema with Aphthous Fever. *Féréol* ........ 671
Silicate of Potassa in Erysipelas. *Alvarenza* ........ 172
Treatment of Acne by Soft Soap. *Hebra-Lallier* ........ 172
Treatment of Alopecia Areata. *Stowers* ........ 173
Use of Induced Currents in Zona. *Faugue* ........ 171
Treatment of Infantile Eczema. *Caspary* ........ 172

### VIII. Syphilis.

Cerebral Syphilis. *Wood* 553
Cerebral Syphilis with Coma. *Mercier* 552
Diagnosis of Pustulo-Crustaceous Syphilide. *Hardy* 951
Effects Produced upon the Blood of Syphilitic Patients by Mercury. *Keyes* 555
Efficacy of Iodide of Potassium in Syphilis. *McSweeny* 63
Influence of Erysipelas on Syphilis. *Deahna* 554
Laryngeal Mucous Patches. *Gambert* 60
Precocious Malignant Syphilides. *Ory* 554
Prognosis in Syphilis. *Hutchinson* 62
Rapidly Malignant Syphilis. *Guibout* 61
Syphilis of the Placenta. *Fitz* 62
Syphilis Variously Treated in Pregnancy. *Weber* 64
Syphilitic Headache and Neuralgia Cured by Small and Repeated Doses of Mercury. *Peter* 669
Syphilitic Laryngeal Vegetations and Pulmonary Gummata. *Maunoir* 60
Syphilitic Liver. *Thompson* 61
Syphilitic Phthisis. *Fournier* 553
Syphilitic Placenta. *Macdonald* 61
Syphiloma of the Tubercula Quadrigemina. *Fiori* 952
Tayuya, a New Antisyphilitic. *Ubicini* 63
Tracheotomy in Extremis for Asphyxia from Syphilitic Laryngitis. *Quise* 951
Transmission of Syphilis between Nurses and Nurslings. *Taylor* 59
Treatment of Papulo-Hypertrophic Syphilide. *Chéron* 63
Treatment of Phagedenic Gangrenous Venereal Sores 63
Treatment of Primary Syphilis by Lewin's Method. *Seeley* 63
Ulcerating Syphilide and Lupus Vulgaris. *Duhring* 59

### REPORTS OF SOCIETIES.

Association of American Medical Editors 723
Association of Representatives of American Medical Congress 725

Chicago Medical Society:
Adenoid Disease 910
Aspergillus Nigricans 913
Calculus of the Ureter 908
Fibroid Tumor of Uterus 914
Hydatidiform Degeneration of the Ovum 917
Occlusion of Larynx by a piece of coal 920
Tumor of the Brain, Calcareous 734

Chicago Society of Physicians and Surgeons:
Rheumatic Arthritis treated with Salicylic Acid 732

Illinois State Medical Society 618
North Central Medical Society 447
Rock River Medical Society 446

### CLINICAL REPORTS.

Chicago Medical College ........ 42, 248
Cook County Hospital ........ 136, 448, 622, 738, 824, 926, 1108
Rush Medical College ........ 36
Mercy Hospital ........ 133, 930
St. Luke's Hospital ........ 538

### BOOK NOTICES.

Balfour, G. W. Diseases of the Heart and Aorta ........ 1116
Balestra, P. Hygiène dans la ville de Rome ........ 568
Baxter, I. H. Statistical Report of the Provost Marshal General's Bureau ........ 939
Brown, J. H. B. The Medical Jurisprudence of Insanity ........ 999
Carpenter, Wm. B. Human Physiology ........ 564
Carter, R. B. Diseases of the Eye ........ 176, 1001
Cohen, J. S. Inhalation in the Treatment of Diseases ........ 1017
Cowperthwait, A. C. Insanity in its Medico-Legal Relations ........ 636
Da Costa, J. M. Medical Diagnosis ........ 1012
Dalton, J. C. Treatise on Human Physiology ........ 83
Darwin, Charles. Insectivorous Plants ........ 181
Denison, Charles. Welfare of Invalids ........ 370
Flint, Austin. Phthisis ........ 357
Gross, S. D. Diseases, Injuries and Malformations of the Bladder ........ 1123
Graham, D. W. Chicago Medical Register ........ 944
Hamilton, Frank. Fractures and Dislocations ........ 374
Heath, Christopher. Minor Surgery and Bandaging ........ 183
Hospital Plans ........ 557
Küss. Lectures on Physiology ........ 182
Lee, H. Lectures on Syphilis ........ 468
Leonard, C. H. A Manual of Bandaging ........ 281
Loring, E. G. Determination of the Refraction of the Eye ........ 648
Lucas-Championnière. Chirurgie Antiseptique ........ 1015
Macnamara, C. Manual of Diseases of the Eye ........ 1006
Parry, J. S. Extra-Uterine Pregnancy ........ 473
Piffard, Henry G. Diseases of the Skin ........ 628
Pozzi, Samuel. De la Valeur de l' Hystérotomie ........ 1003
Reeves, C. E. Consumption in Australia ........ 1016
Rutherford, Wm. Practical Histiology ........ 182
Sayre, L. A. Lectures on Orthopedic Surgery ........ 1008
Seguin, E. C. American Clinical Lectures ........ 279
Smith, J. L. Diseases of Infancy and Childhood ........ 934
Smith, R. D. O. Odorless Water-closets ........ 640
Spratt, E. Synopsis of the Symptoms of Gout of the Heart ........ 476
Transactions of the Colorado Territorial Medical Society ........ 373
Transactions of the Pathological Society of Philadelphia. Vol. V ........ 1011
Transactions of the Illinois State Medical Society, 1875 ........ 1013

Taylor, R. W. Syphilitic Lesions in Infants and Children............ 378
Wagstaffe, W. W. The Student's Guide to Human Osteology........1016
Winckel, F., M.D. Pathology and Treatment of Children............ 642
Wood, H. C. Therapeutics..................................... 174
Woodworth, J. M. Government Report on Cholera.................. 72
Ziemssen, H. von. Cyclopædia of the Practice of Medicine. Vol. III. 468

### OBITUARY.

Sherman, Julian S., A.M., M.D.................................1032

### BOOKS AND PAMPHLETS RECEIVED.

The Anatomy of the Head, with six Lithographic Plates, representing frozen sections of the head. By Thos. Dwight, M.D., etc. 1876.

Ziemssen's Cyclopædia of Practical Medicine. Volume VI. Diseases of the Circulatory System.

Observations on the Diseases of the Rectum. By T. B. Curling, F.R.S., etc. Fourth edition, revised and enlarged. 1876.

Chemia Coartata; or the Key to Modern Chemistry. By A. H Kollmyer, A.M., M.D., etc. 1876.

A Treatise on the Theory and Practice of Medicine. By Jno. Syer Bristowe, M.D., etc. Edited with notes by J. H Hutchinson, M.D., etc. 1876.

Public Libraries in the United States of America, their history, condition, and management. Special report, Department of the Interior, Bureau of Education. Part I.

Epitome of Skin Diseases, with formulæ. For Students and Practitioners. By Tilbury Fox, M.D., F.R.C.P., etc., and T. C. Fox, B.A., M.R.C.S. Henry C. Lea. 1876.

Principles of Human Physiology. By Wm. B. Carpenter, M.D., etc. Edited by Henry Power, M.B., etc. A new American edition, from the eighth revised and enlarged English edition, with notes and additions. By Francis G. Smith, M.D., etc. Henry C. Lea. 1876.

Transactions of the Texas State Medical Association. Eighth Annual Session, 1876.

Salicylic Acid ; the experience of Maine Physicians in its use. By F. H. Gerrish, M.D. 1876.

The Hypertrophied Prostate. By R. F. Weir, M.D., etc. Being No. VIII., of Vol. II., of "American Clinical Lectures."

Life: What do we know about it? An Essay read before the Chicago Literary Club, May, 1876. By H. A. Johnson, M.D.

The Treatment of Auteflexions of the Uterus. By Ely Van De Warker, M.D. 1876.

Case of Large Cerebral Tumor, without Optic Neuritis, and with left Hemiplegia and Imperception. By J. Hughlings Jackson, M.D., F.R.C.P.

A Case of Double Optic Neuritis, without Cerebral Tumor. By J. Hughlings Jackson, M.D., F.R.C.P.

Points in the Surgery of Childhood. By J. H. Pooley, M.D., etc. Being No. IX., of Vol. II., of "A Series of American Clinical Lectures."

Hygeia, a City of Health. A Presidential Address, delivered before the Health Department of the Social Science Association, Oct. 1875. By B. W. Richardson, M.D., F.R.S. 1876.

Johns Hopkins Hospital. Reports and Papers relating to Construction and Organization. No. 2, with 14 Plates. By Jno. S. Billings, M.D. The Plans and Plates by J. R. Niernsée, Architect.

Transactions of the Medical Society of the State of Pennsylvania, at its 27th Annual Session, held at Philadelphia, May 31, and June 1, 1876. Vol. XI., Part I.

Transactions of the Medical Society of the State of California, during the years 1875–6.

Specialists and Specialties in Medicine. Address. By H. M. Henry, M.A., M.D., etc.

A Lecture on Specialism in Medicine. By E. D. Forée, M.D.

Vaginal Ovariotomy. By Clifton E. Wing, M.D.

The Operation for Stone as observed in some of the London Hospitals, together with a report of cases from private practice. By A. Van Derveer, M.D., etc.

---

The following exchanges were received during the year:

Allgemeine Medizin. Centralzeitung.
American Journal of Insanity.
American Journal of Medical Sciences.
American Journal of Microscopy.
American Journal of Obstetrics.
American Journal of Pharmacy.
American Medical Weekly.
American Practitioner.
American Psychological Journal.
Anales de Ciençias Médicas, Madrid.
Anales de la Asociacion, Larrey, Mex.
Annales de Dermatologie et de Syphilig.
Annali Clinici della Ospedale Incurabili.
Archiv der Heilkunde.
Archives Générales de Médecine.
Archives of Clinical Surgery.
Archives of Electrology.
Archives of Dermatology.
Archives of Science.
Atlanta Medical and Surgical Journal.
Boston Journal of Chemistry.
Boston Medical and Surgical Journal.
Braithwaite's Retrospect.
Buffalo Medical and Surgical Journal.
Canada Lancet.
Canada Medical Journal.
Canada Medical and Surgical Journal.
Canadian Journal of Medical Science.
Centralblatt fuer Chirurgie.
Charleston Medical and Surgical Journal.
Chicago Journal of Nervous Diseases.

Cincinnati Lancet and Observer.
Detroit Review of Medicine and Pharmacy.
Deutsche Mediz. Wochenschrift.
Edinburgh Medical and Surgical Journal.
France Médicale.
Galveston Medical Journal.
Gazette Médicale.
Gazette Obstétricale et Gynécologique.
Gazzetta Medica Italiana, Lombardia.
Gazzetta Medica Delle Calabrie.
Giornale della R. Accademia di Medicina.
Giornale Veneto di Scienze Medic.
Indiana Journal of Medicine.
Journal de la Physiologie.
Journal de Méd. et de Chiruag. Pratiques.
Journal of the British Association.
London Lancet.
London Medical Times and Gazette.
Louisville Medical News.
L'Union Médicale.
L'Union Médicale du Canada.
Lyon Médical.
Melbourne Medical Record.
Memorabilien.
Nashville Journal of Medicine and Surgery.
New Orleans Medical and Surgical Journal.
New Remedies.
New York Medical Journal.
New York Medical Record.
Obstetrical Journal.
Ohio Medical Recorder.
Ohio Medical and Surgical Journal.
Oregon Medical Journal.
Oregon Medical and Surgical Reporter.
Peninsular Journal of Medicine.
Pharmacist.
Philadelphia Medical and Surgical Reporter.
Philadelphia Medical Times.

Polytechnic Review.
Progrès Médical.
Recueil d'Ophthalmologie.
Revista Medico-Quirurgica, Buenos Ayres.
Richmond Medical Journal.
Sanitarian.
Savannah Journal of Medicine.
Southern Medical Record.
St. Louis Clinical Record.
St. Louis Medical and Surgical Journal.
Student's Journal and Hospital Gazette.
The Clinic.
The Doctor.
The Practitioner, London.
The Western Lancet.
The West Virginia Medical Student.
Vierteljahresschrift fuer Dermatologie und Syphil.
Virginia Clinical Record.
Wiener Medizin. Chirurgisches Centralblatt.
Wiener Medizin. Wochenschrift.

### ANNOUNCEMENTS FOR THE MONTH.

**MONDAYS.** SOCIETIES.

*Mondays, Dec.* 4 *and* 18.—Chicago Medical Society. Regular meetings.
*Mondays, Dec.* 11 *and* 25.—Chicago Soc. of Phys. and Surgeons. Regular meetings.

CLINICS. *Every Monday.*

At Eye and Ear Infirmary, 2 P. M.—Prof. Holmes.
At Central Dispensary (Wood and Harrison sts.), 2 P.M. *Gynecological*—Dr. Adolphus.
At Mercy Hospital—2 P. M., *Surgical*, Prof. Andrews.
At Rush College—2¼ P. M., *Medical*, Dr. Bridge.
At Chicago College, 2 P. M *Gynecological*—Prof. Merriman.

LECTURES. *Every Monday.*

At Rush Medical College (Harrison and Wood sts.)—9½ to 12½ o'clock, Profs. Freer, Lyman and Powell; 4 to 6, Profs. Gunn and Haines. At Chicago College—8½ to 12½, Profs. Jewell, Hyde, Hatfield and Bond; 3 to 6, Profs. Nelson and Davis, Quine and Andrews, and Roler. At Woman's College—8½ to 11½, Profs. Hotz, Marguerat and Earle; 3 to 5, Profs. Paoli and Stevenson.

**TUESDAYS.** SOCIETIES.

*Tuesday, Dec.* 12.—Academy of Science. Regular meeting at 8 P. M. (263 Wabash av.)

CLINICS. *Every Tuesday.*

At Eye and Ear Infirmary—2 P. M., Prof. Jones.
At County Hospital—2 P. M., *Medical*, Prof. Ross. 3 P. M., *Surgical*, Prof. Powell.
At Mercy Hospital—2 P. M., *Medical*, Prof. Hollister.
At Chicago College—2 P. M., *Gynecological*, Prof. Roler.
At Central Dispensary—2, *Surgical*, Dr. Graham.

LECTURES. *Every Tuesday.*

At Rush College—9½ to 12½, Profs. Miller, Allen and Parkes; 4 to 6, Profs. Gunn and Haines. At Chicago College—8½ to 12½, Profs. Jewell, Quine, Merriman and Bond; 3 to 6, Profs. Johnson and Nelson, Andrews and Hatfield, and Byford. At Woman's College—9 to 12, Profs. Bartlett, Bogue and Fitch; 3 and 5, Profs. Thompson and Macdonald.

**WEDNESDAYS.** CLINICS. *Every Wednesday.*

At Eye and Ear Infirmary—2 P. M., Prof. Holmes.
At County Hospital—2 P. M., *Gynecological*, Prof. Fitch; 2 P. M., *Ophthalmological*, Dr. Montgomery.
At Chicago College—2 P. M., *Gynecological*, Prof. Nelson.
At Mercy Hospital—2 P. M., *Ophthalmological*, Prof. Jones.
At Central Dispensary—3 P. M., *Dermatological*, Dr. Maynard.

LECTURES. *Every Wednesday.*

At Rush College—9½ to 12½, Profs. Freer, Allen and Parkes; 4 to 6, Profs. Etheridge and Haines. At Chicago College—8½ to 12½, Profs. Hyde, Isham, Hatfield and Bond; 3 to 6, Profs. Davis and Curtis, Quine and Jones, and Roler. At Woman's College—9 to 12, Profs. Marguerat, Curtis and Blake; 3 to 6, Paoli, Graham and Stevenson.

**THURSDAYS.** CLINICS. *Every Thursday.*

At Eye and Ear Infirmary—2 P. M., Prof. Hotz.
At Mercy Hospital—2 P. M., *Medical*, Prof. Davis.
At Rush College—2 P. M., *Medical*, Prof. Ross; 3 P. M., *Diseases of the Nervous System*, Prof. Lyman.
At Chicago College—2 P. M., *Gynecological*, Prof. Merriman.
At Central Dispensary—2 P.M., *Surgical*, Dr. Graham.

LECTURES. *Every Thursday.*

At Rush College—9½ to 12½, Profs. Miller, Allen and Parkes; 4 to 6, Profs. Gunn and Etheridge. At Chicago College—8½ to 12½, Profs. Hollister, Isham, Merriman and Bond; 3 to 6, Profs. Johnson and Nelson, Andrews and Hatfield, and Byford. At Woman's College—9 to 12, Profs. Dyas, Bogue and Fitch; 5, Prof. Macdonald.

**FRIDAYS.** SOCIETIES.

*Friday, Dec.* 8.—State Microscopical Society of Illinois. Regular meeting, 8 P. M.

CLINICS. *Every Friday.*

At County Hospital—2 P. M., *Medical*, Prof. Ross; 3 P. M., *Surgical*, Prof. Powell.
At Mercy Hospital—2 P. M., *Medical*, Prof. Davis.
At Chicago College—2 P. M., *Gynecological*, Prof. Roler.
At Central Dispensary—2 P. M., *Gynecological*, Dr. Adolphus.

LECTURES. *Every Friday.*

At Rush College—9½ to 12½, Profs. Freer, Allen and Parkes; 4 to 6, Profs. Gunn and Holmes. At Chicago College—8½ to 12½, Profs. Hollister, Isham, Hatfield and Bond; 3 to 6. Profs. Davis and Curtis, Jones and Quine, and Roler. At Woman's College—9 to 12, Profs. Bartlett, Marguerat and Curtis; 5, Prof. Stevenson.

**SATURDAYS.** CLINICS. *Every Saturday.*

At Chicago College—2 P. M., *Surgical*, Prof. Andrews or Isham; *Gynecological*, Prof. Nelson; 3 P. M., *Medical*, Prof. Johnson.
At Rush College—2 P. M., *Surgical*, Prof. Gunn.

LECTURES. *Every Saturday.*

At Rush College—8½ to 12½, Profs. Lyman, Miller, Etheridge and Parks. At Chicago College, 8½ to 11½, Profs. Hollister, Quine and Hyde; 3 to 6, Profs. Nelson and Johnson, Andrews and Quine and Byford. At Woman's College—9 to 11, Profs. Earle and Fitch; 3 and 5, Profs. Paoli and Macdonald.

At all the above named Clinics visiting regular practitioners are, we believe, admitted.
At the South Side Dispensary (Chicago College) there are six daily special Clinics, for sections of the classes of the Chicago College.

